# P04.17. Adverse effects of homeopathy, what do we know? A systematic review

**DOI:** 10.1186/1472-6882-12-S1-P287

**Published:** 2012-06-12

**Authors:** T Stub, F Musial, T Alræk

**Affiliations:** 1 University of Tromsø, Tromsø, Norway

## Purpose

Homeopathy has few legal regulations acting as gatekeepers. The remedies may be in widespread use despite unclear mechanism of effect and safety assessment. Uncontrolled studies of homeopathic practise document consistently strong therapeutic effects and sustained patient satisfaction however, cases of adverse effects have also been reported. According to homeopathic theory transient worsening of patients symptoms (aggravations), are understood as a wanted reaction to the medication. To date, systematic information is lacking on how commonly adverse effects and homeopathic aggravations are reported in RCTs, observational studies and surveys.

## Methods

A systematic review addressing this topic was undertaken. Twelve electronic databases were searched.

## Results

Twenty-seven RCTs, 26 observational studies and 4 surveys, with a total of 28,917 participants were included in this review. The methodological quality assessed according to the Cochrane handbook for RCTs and STROBE checklist for observational studies and surveys was high. Twenty-one percent of the RCTs, 36.5% of the observational studies and 16% of the surveys reported cases of adverse effects such as gastro-intestinal disorders, headache and dermatitis. Of these, 14% were reported as serious events. Eighteen percent of the RCTs, 36.5% of the observational studies and 8% of the surveys reported homeopathic aggravations which were mostly reported as intensifications of the patient’s symptoms.

## Conclusion

In order to prevent serious events as a consequence of homeopathic treatment, the identification of an unwanted adverse event is of critical importance. A differentiation of adverse events and homeopathic aggravations, which is accepted as a concept in homeopathy, should be a part of a reporting system where risk and safety are assessed. This is of particular significance in a treatment system like homeopathy, which is in most European countries regulated as an alternative treatment and as such not included in the supervision system of health care.

